# Estrogen prevented gingival barrier injury from *Porphyromonas gingivalis* lipopolysaccharide

**DOI:** 10.1128/iai.00410-24

**Published:** 2025-02-20

**Authors:** Fangting Huang, Zhifei Su, Fangjie Zhou, Yajie Wu, Jiyao Li, Biao Ren

**Affiliations:** 1 State Key Laboratory of Oral Diseases, National Center for Stomatology, National Clinical Research Center for Oral Diseases, West China School of Stomatology, Sichuan University 616175 , Chengdu, Sichuan, China; 2 Department of Preventive Dentistry, Hospital of Stomatology, Guanghua School of Stomatology, Sun Yat-Sen University 540445 , Guangzhou, Guangdong, China; 3 Department of Cariology and Endodontics, West China School of Stomatology, Sichuan University 617366 , Chengdu, Sichuan, China; Georgia Institute of Technology, Atlanta, Georgia, USA

**Keywords:** estrogen, periodontitis, gingiva, epthilium, lipopolysaccharide, tight junction proteins

## Abstract

The postmenopausal population usually suffers from more severe periodontal disease than non-menopausal women due to the decrease and low levels of estrogen, especially β-estradiol (E2). While additional estrogen therapy can effectively relieve alveolar bone resorption, this suggests that estrogen has played an important role in the development of periodontitis. The integrity of the gingival epithelial barrier plays a key role in protecting gingival tissue from inflammatory injury caused by pathogens. However, it remains unclear whether estrogen can maintain the integrity of the gingival epithelial barrier to reduce inflammatory injury. Here, using an infection model established with *Porphyromonas gingivalis* lipopolysaccharide (LPS) in human gingival epithelial cells (hGECs) and ovariectomized or Sham mice, we assessed the protective effect of estrogen on the gingival barrier using qPCR, western blotting, immunohistochemistry, and transcriptome analysis. The results showed that estrogen restored epithelial barrier function to inhibit *P. gingivalis*-LPS invasion and further downregulate the inflammatory reaction (*P* < 0.05) by upregulating expressions of tight junction proteins (such as JAM1 and OCLN) at mRNA and protein levels in both hGECs and ovariectomized or Sham mice (*P* < 0.05). Estrogen also protected against alveolar bone resorption and preserved barrier integrity in both ovariectomized and Sham mice (*P* < 0.05). In conclusion, E2 prevented the progression of gingival epithelial barrier damage and inflammation induced by *P. gingivalis*-LPS by increasing the expression of tight junction proteins. The protective effect of estrogen on gingival epithelial barrier injury highlighted its potential application in treating periodontitis and inflammatory diseases involving epithelial barrier dysfunction.

## INTRODUCTION

Periodontitis, a chronic inflammatory disease, affects >40% of adults and enormously reduces quality of life due to alveolar bone resorption and tooth loss (1, 2). Estrogen deficiency is a risk factor for periodontitis (3–5). Estrogen, especially β-estradiol (E2), gradually decreases and remains at a low concentration level in the postmenopausal population, leading to more severe periodontal disease than that in non-menopausal women (6–8). Some clinical and animal studies found that supplementary E2 treatment has the potential to protect alveolar bone resorption and inhibit periodontitis progression (9–11) and indicated the key roles of E2 in the development of periodontitis and the functions of E2 to maintain the homeostasis of alveolar bone and resist host inflammatory responses (12). However, periodontitis is a microbiota-driven inflammatory disease, and there is an urgent need for further investigation that whether E2 treatment would promote the resistance of gingival tissue or cells to periodontal pathogens (13).

The gingival epithelial barrier, consisting of the gingival epithelium and various transmembrane proteins, provides the first line of defense against the invasion of periodontal pathogens (14). Periodontal pathogens and their virulence factors were harmful to the structural integrity of the epithelial barrier by decreasing the expression of transmembrane proteins in gingival epithelium cells, such as occludin (OCLN) and junctional adhesion molecule 1 (JAM1) (15–17). Occludin is a four-transmembrane protein with signal transduction functions. JAM1 is also a four-transmembrane protein and a member of the immunoglobulin superfamily of proteins. These junction proteins play key roles in cell-cell adhesion, vascular permeability, pathogens prevention, and immune cell trafficking to maintain the integrity of epithelial barriers. However, when the epithelial barrier is damaged, it facilitates the progression of inflammation and periodontitis (18). Recent advances in understanding the functions of E2 in HIV infection, inflammatory bowel disease, and eosinophilic esophagitis indicated that E2 contributed to the restoration of the epithelial barrier integrity damaged by different pathogens (19–21). Importantly, the discovery of the estrogen receptor β in human gingival epithelial cells (hGECs) provided the possibility that E2 affects the behavior of gingival epithelial cells (22–24). However, the effect of E2 on protecting the host from periodontal pathogens challenge by impacting the gingival epithelial barrier remains unclear.

*Porphyromonas gingivalis*, a key opportunistic pathogen in periodontitis, showed the ability to impair the epithelial barrier (25), while its lipopolysaccharide (LPS), the pivotal virulence factor, showed a stimulatory effect on the disruption of the epithelial barrier (26), indicating that the damage on the gingival epithelial barrier of *P. gingivalis* is critical in its infectious process to cause periodontitis.

Accordingly, this study aimed to investigate the protective effect and mechanisms of E2 against the gingival epithelial barrier injury of hGECs caused by *P. gingivalis*-LPS and to further explore the effect of E2 on the gingival epithelial barrier and alveolar bone resorption in an ovariectomized and Sham mouse model of periodontitis.

## RESULTS

### E2 inhibited the expression and production of inflammatory cytokines in *P. gingivalis*-LPS-treated hGECs

**Fig 1:**
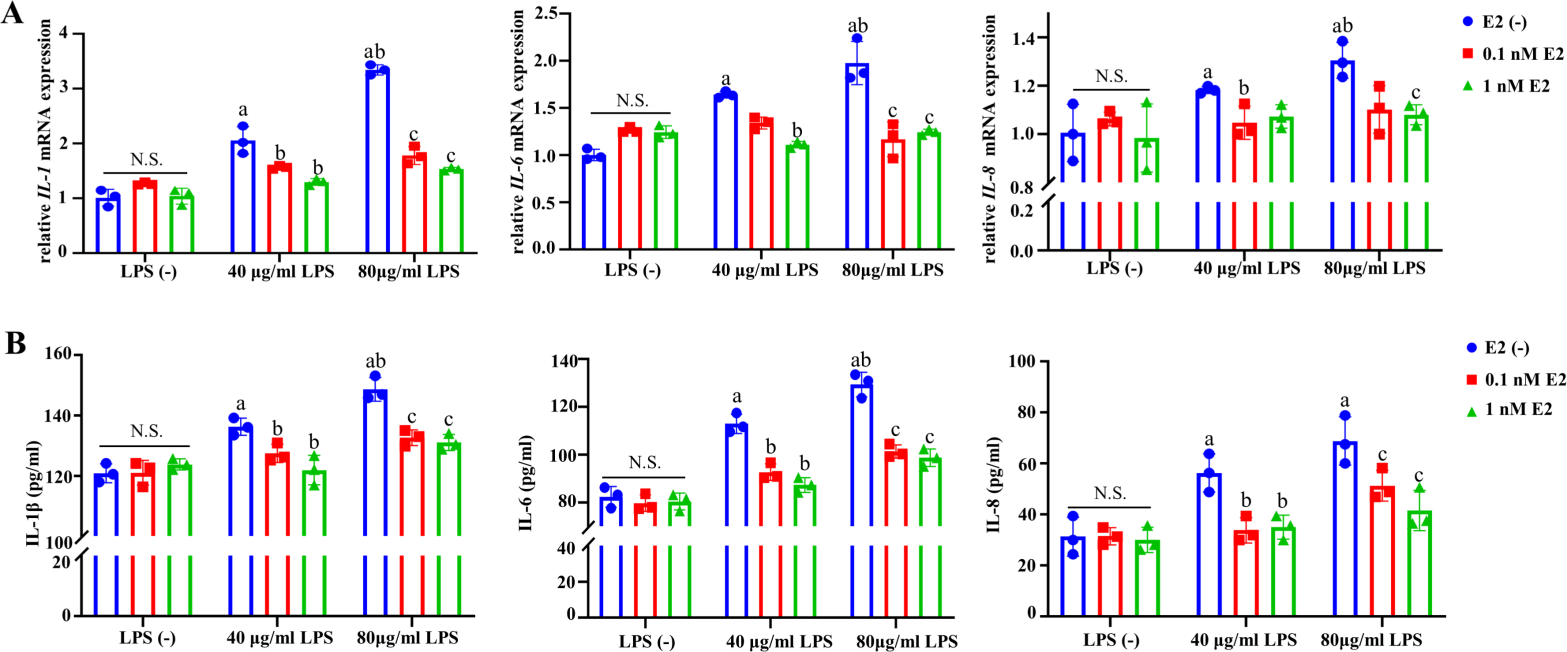
Anti-inflammatory properties of E2 in *P. gingivalis*-LPS-treated hGECs. (**A**) mRNA expression levels of IL-1 (left panel), IL-6 (middle panel), and IL-8 (right panel) determined by quantitative real-time PCR. (**B**) Protein expression levels of IL-1 (left panel), IL-6 (middle panel), and IL-8 (right panel) determined by ELISA. Data represent means ± SDs (*n* = 3). a: *P* < 0.05 compared with the LPS (−)/E2 (−) group. b: *P* < 0.05 compared with the 40 µg/mL LPS/E2 (−) group. c: *P* < 0.05 compared with the 80 µg/mL LPS/E2 (−) group. N.S.: no statistical significance.

To first assess the effect of E2 on *P. gingivalis*-LPS-treated hGECs, the expression of inflammatory cytokines was detected. *P. gingivalis*-LPS significantly increased the mRNA expressions of inflammatory cytokines (IL-1β, IL-6, and IL-8) at dose-dependent manner compared with the LPS(−)/E2(−) group. Neither 0.1 nM nor 1 nM E2 affected the expressions of inflammatory cytokines, but E2 significantly decreased their expressions induced by *P. gingivalis*-LPS (*P* < 0.05; Fig. 1A). Similarly, the enzyme-linked immunosorbent assay (ELISA) results revealed that *P. gingivalis*-LPS also significantly increased the productions of IL-1β, IL-6, and IL-8, while both 0.1 and 1 nM E2 significantly inhibited their productions although the single treatment of E2 showed no effects (Fig. 1B). These findings indicated that E2 directly inhibited the inflammatory induction of *P. gingivalis*-LPS in gingival epithelial cells.

### E2 restored the transcriptomic and epithelial barrier-related genes’ expression shifts caused by *P. gingivalis* LPS

**Fig 2:**
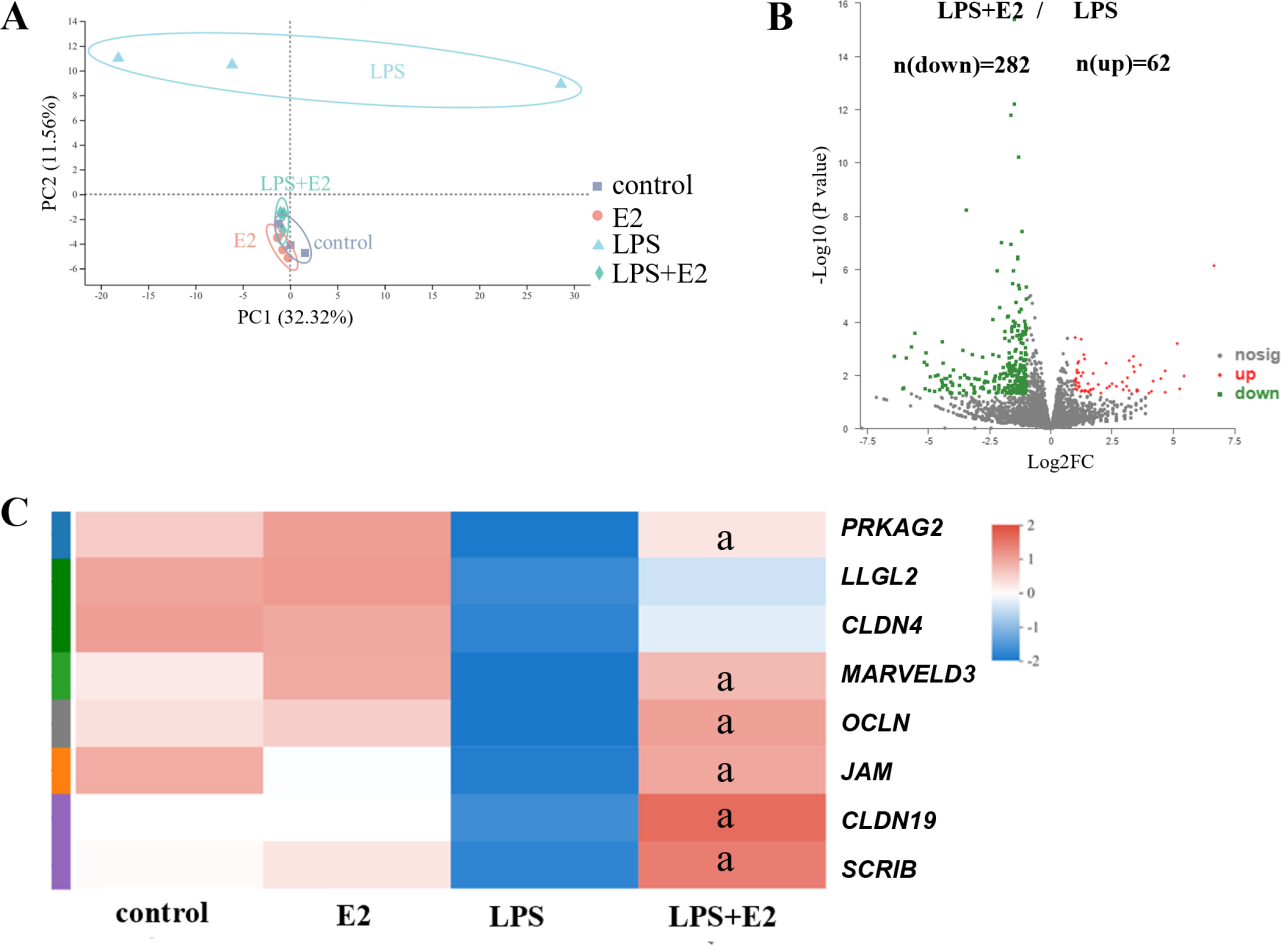
Protective effect of E2 on epithelial barrier injury revealed by RNA sequencing. (**A**) PCA of transcriptome profiling of hGECs. (**B**) Volcano plot of DEGs between the LPS + E2 group and LPS group. (**C**) Heatmap of the expression levels of epithelial barrier-related genes in each group. a: *P* < 0.05 compared with the LPS group.

To reveal the mechanisms that how the E2 impacts the inflammatory induction caused by *P. gingivalis*-LPS, we then analyzed the transcriptome profiles of hGECs from the control, *P. gingivalis* LPS treatment, E2 treatment, and *P. gingivalis-LPS*-LPS + E2 treatment groups. The principal component analysis (PCA) result showed that LPS treatment induced remarkable changes in the transcription status of hGECs, while E2 treatment showed no significant impact compared with the control group (Fig. 2A). Importantly, E2 treatment restored the transcriptomic changes caused by *P. gingivalis* LPS as the transcription status from LPS + E2 group was very similar with the control and E2 treatment groups but quite different from the LPS treatment group (Fig. 2A). Accordingly, the Kyoto Encyclopedia of Genes and Genomes (KEGG) enrichment analysis showed that upregulated genes (LPS compared to control group) were mostly involved in the inflammatory response (Fig. S1A) in line with the inflammatory induction of *P. gingivalis* LPS (Fig. 1). Moreover, there were 344 differentially expressed genes (DEGs) between the LPS + E2 and LPS groups, including 62 upregulated genes and 282 downregulated genes (Fig. 2B), and these downregulated genes (LPS + E2 compared to LPS group) were enriched in the inflammatory response related pathways (Fig. S1B). The expressions of cytokines participating in the inflammatory response of hGECs were significantly decreased in the LPS + E2 group compared to the LPS group (Fig. S2), suggesting that E2 treatment inhibited the inflammatory response caused by *P. gingivalis* LPS. It was noteworthy that LPS significantly decreased the expressions of the key genes involved in epithelial barrier formation, but most of them (7/8) were significantly restored in the LPS + E2 group compared with the LPS group (Fig. 2C). The increase of epithelial barrier function and decrease of inflammatory response of E2 against the opposite actions of *P. gingivalis* LPS suggested that E2 might prevent the epithelial barrier damage from *P. gingivalis* LPS to block the further inflammatory responses.

### E2 recovered the damaged barrier function in *P. gingivalis*-LPS-treated hGECs

**Fig 3:**
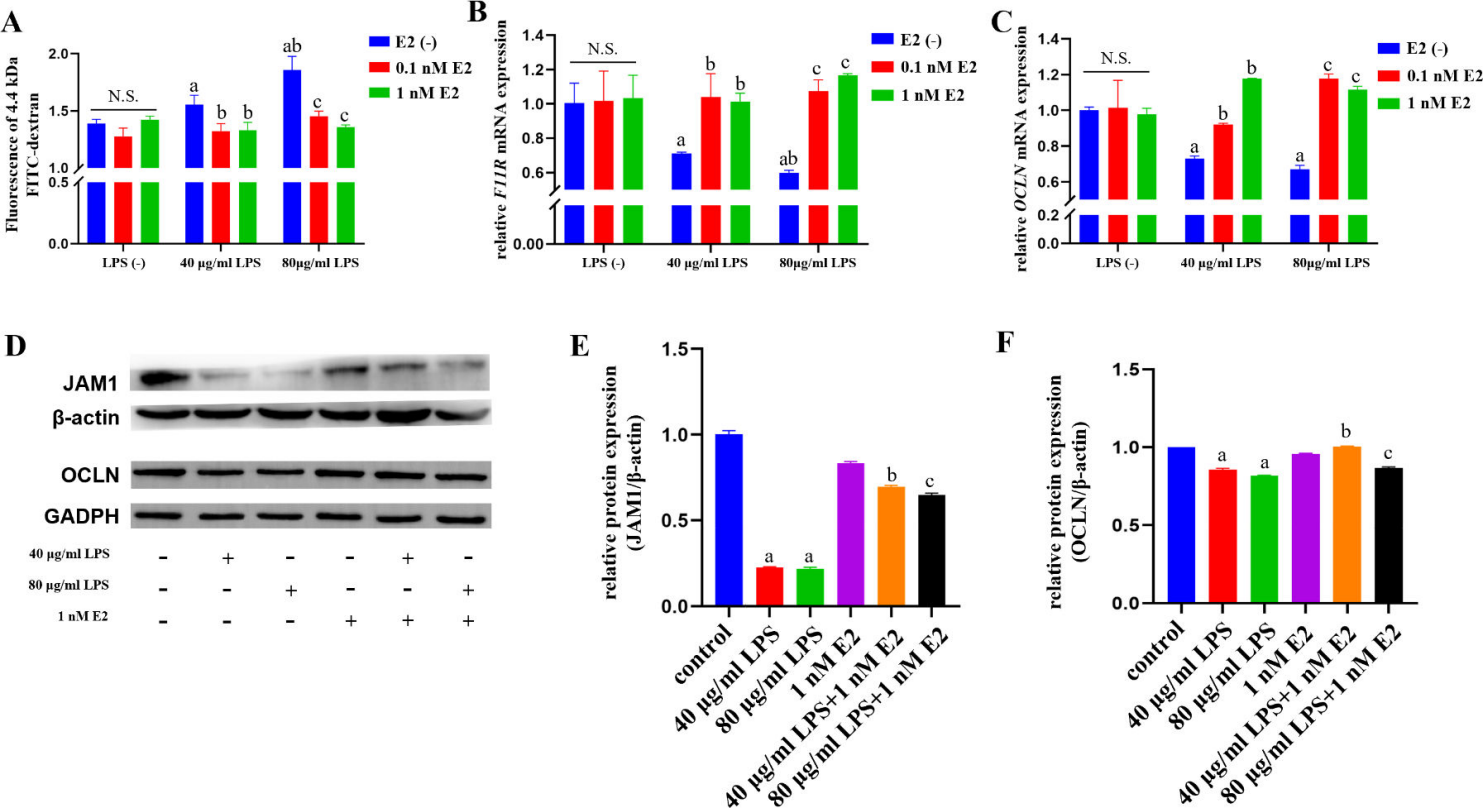
Protective effect of E2 against *P. gingivalis*-LPS-induced epithelial barrier injury in hGECs. (**A**) the fluorescence intensity of FITC-dextran. (**B and C**) mRNA expression levels of *F11R* (**B**) and *OCLN* (**C**) determined by quantitative real-time PCR. (**D**) Representative images of the protein expression levels of JAM1 and OCLN determined by western blotting. (**E and F**) Expression levels of JAM1 (**E**) and OCLN (**F**) analyzed by ImageJ. Data represent means ± SDs (*n* = 3). a: *P* < 0.05 compared with the LPS (−)/E2 (−) group. b: *P* < 0.05 compared with the 40 µg/mL LPS/E2 (−) group. c: *P* < 0.05 compared with the 80 µg/mL LPS/E2 (−) group. N.S.: no statistical significance.

To further confirm the protective capability of E2 on the epithelial barrier, we then directly measured the permeability of hGECs monolayer barrier. Compared with the LPS(−)/E2(−) group, LPS significantly increased the level of fluorescein isothiocyanate-conjugated dextran (FITC-dextran) penetration (*P* < 0.05; Fig. 3A), indicating the epithelial barrier damage caused by *P. gingivalis*-LPS, while E2 significantly reversed the effects and made the epithelial barrier permeability back to the levels from control group (*P* < 0.05; Fig. 3A). To further identify how the LPS damaged and E2 protected the epithelial barrier, we detected the expressions of two important tight junction proteins, JAM1 and OCLN, which played an important role in epithelial barriers. LPS treatment significantly decreased the mRNA expressions of JAM1 (Fig. 3B) and OCLN (Fig. 3C) and their proteins (Fig. 3D through F), in line with their expressions from the transcriptome analysis (Fig. 2C). Both 0.1 nM and 1 nM E2 could restore their expressions in mRNA or protein levels (*P* < 0.05; Fig. 3B through F), indicating that E2 recovered the epithelial barrier function by restoring the expressions of epithelial tight junction proteins that were downregulated by *P. gingivalis* LPS.

### E2 inhibited the inflammatory reaction by protecting the epithelial barrier function in hGECs

**Fig 4:**
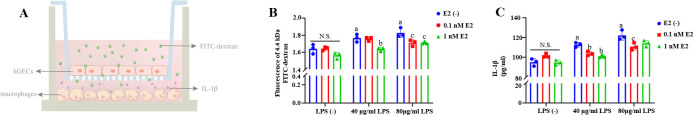
Inhibitory effect of E2 on the inflammatory reaction by protecting the epithelial barrier function in hGECs. (**A**) The model was used for the transport and cytokine assays. The FITC-dextran was added to the upper compartment, and the fluorescence signal in the bottom compartment was determined after 1 h to test the barrier permeability. Additionally, to examine pathogen cell permeability, *P. gingivalis* LPS was used as a transport substance and added to the upper compartment. Cytokine production from macrophages was collected and measured in the bottom compartment. (**B**) The fluorescence intensity of FITC-dextran in the lower compartment. (**C**) Protein expression levels of IL-1β in macrophages determined by ELISA. Data represent means ± SDs (*n* = 3). a: *P* < 0.05 compared with the LPS (−)/E2 (−) group. b: *P* < 0.05 compared with the 40 µg/mL LPS/E2 (−) group. c: *P* < 0.05 compared with the 80 µg/mL LPS/E2 (−) group. N.S.: no statistical significance.

To further investigate whether the recovery of epithelial barrier function by E2 would directly reduce the inflammatory induction from *P. gingivalis*-LPS, we performed transport and cytokine assays in a transwell system (Fig. 4A). *P. gingivalis* LPS induced a higher transport level of the epithelial barrier (increased FITC-dextran level in the lower compartment; Fig. 4B), in line with that in Fig. 2A. In addition, a higher inflammatory reaction was observed in macrophages (increased IL-1β in the lower compartment; Fig. 4C). Importantly, additional E2 treatment decreased the transport level compared to the LPS group and further inhibited IL-1β expression in macrophages (Fig. 4B and C). In summary, E2 inhibited *P. gingivali*s LPS invasion and inflammatory reactions, indicating that supplementary E2 treatment inhibited the progression of periodontitis by enhancing epithelial barrier resistance to pathogens or metabolites invasion.

### E2 protected gingival epithelial barrier against *P. gingivalis* LPS to restore alveolar bone resorption in both sham and ovariectomized (OVX) mice

**Fig 5:**
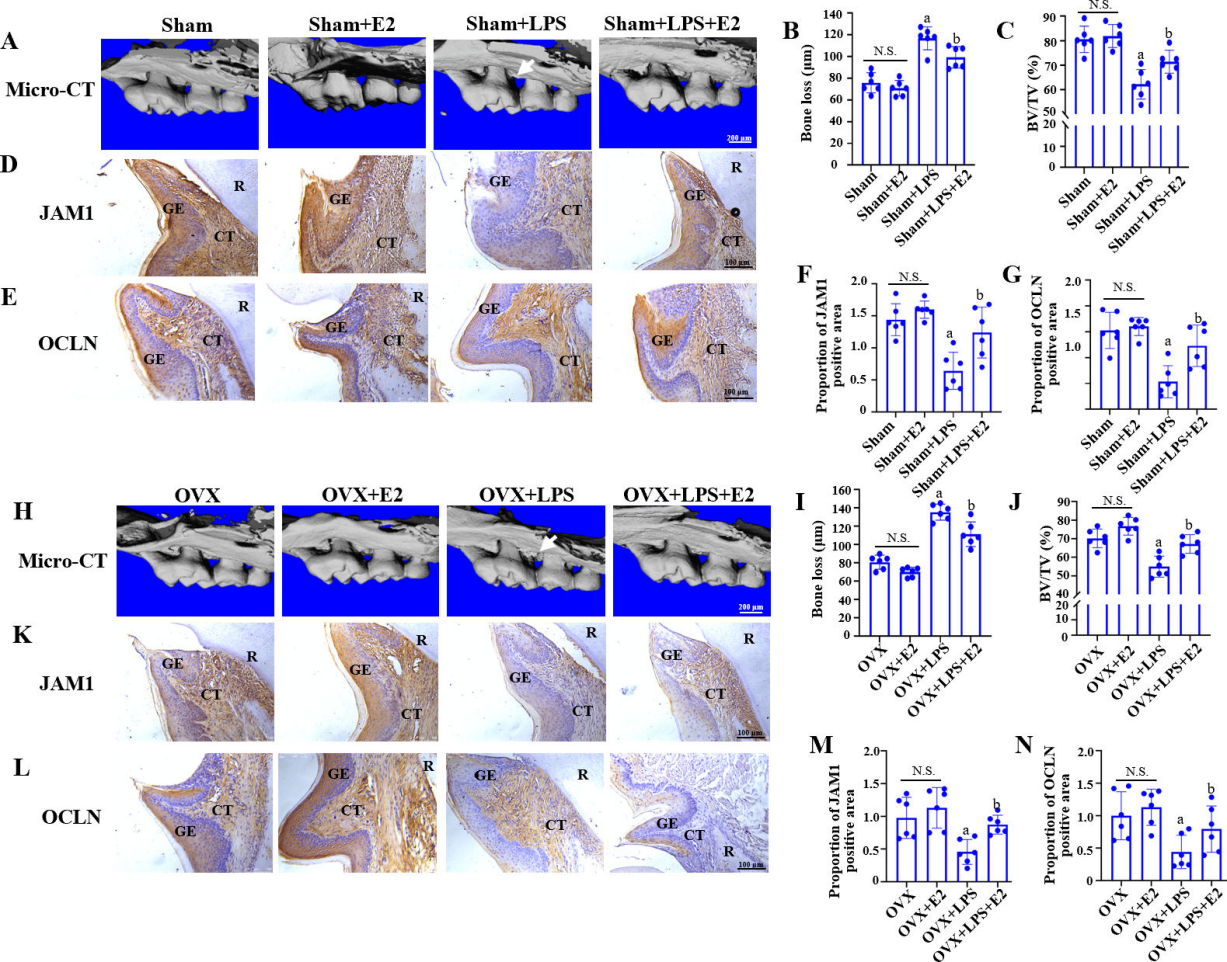
Protective effect of E2 against *P. gingivalis*-LPS-induced gingival epithelial barrier destruction and alveolar bone resorption *in vivo*. (**A and H**) Micro-computed tomography of the maxillary first molars. White arrow: bone resorption region in the LPS injection group (bar = 200 µm). (**B and C, I and J**) Quantitative analysis of bone loss (**B and I**) and bone volume/tissue volume (BV/TV, **C and J**). (**D and E, K and L**) Immunohistochemical staining of JAM1 (**D and K**) and OCLN (**E and L**) in periodontal tissue. R: root. GE: gingival epithelial. CT: connective tissue. (bar = 100 µm). (**F and G, M and N**) Quantitative analysis of JAM1 (**F and M**)- and OCLN (**G and N**)-positive areas. Data represent means ± SDs (*n* = 6). a: *P* < 0.05 compared with the Sham/ OVX group. b: *P* < 0.05 compared with the Sham + LPS/OVX + LPS group. N.S.: no statistical significance.

We then established the Sham and OVX mice models to confirm the gingival epithelial barrier protective effect of E2 against *P. gingivalis* LPS. According to the micro-computed tomography (micro-CT) analysis, the LPS significantly increased bone loss and decreased bone volume/tissue volume (BV/TV) ratio compared to that from the control groups in both Sham (Fig. 5A through C) and OVX mice (Fig. 4H through J), while the supplementary E2 treatment significantly rescued the bone loss and the decreased BV/TV ratio caused by *P. gingivalis* LPS in both Sham (Fig. 5A through C) and OVX mice (Fig. 5H through J), indicating that E2 protected host against *P. gingivalis*-LPS-induced alveolar bone resorption.

Next, we used immunohistochemistry (anti-JAM1 and anti-OCLN) to decipher the effect of E2 on the gingival epithelial barrier. We found that the JAM1- or OCLN-positive area was significantly decreased in the LPS injection group compared to the Sham/ OVX group, while supplementary E2 treatment enhanced the expression of JAM1 and OCLN in the Sham + LPS + E2 and OVX + LPS + E2 group (Fig. 4D through G and K through N). In summary, we found experimental evidence that highlights the protective effect of E2 on the gingival epithelial barrier and alveolar bone resorption *in vivo*.

## DISCUSSION

LPS, an important pathogenic factor of *P. gingivalis*, induces the inflammatory response in gingival epithelial cells (27, 28). Therefore, we used *P. gingivalis* LPS stimulation to construct an infective hGECs model and found that LPS stimulation caused an inflammatory response and damaged barrier function, including increased inflammatory cytokines (IL-1, IL-6, and IL-8) and decreased tight junction-related factors (JAM1 and OCLN). In addition, we also found that local injection of *P. gingivalis LPS* led to significant periodontitis (increased bone loss and decreased BV/TV ratio) and remarkable epithelial barrier destruction (decreased JAM1/OCLN positive area in the gingival epithelium) in both Sham and OVX mice. Taken together, our data confirmed that *P. gingivalis* LPS exerted a powerful effect on the induction of inflammation and destruction of the epithelial barrier *in vitro* and *in vivo*.

The *P. gingivalis*-LPS-induced inflammatory response played a crucial role in the progression of periodontitis (29). Previously, Li et al. found that psoralen, an agonist to the estrogen receptor, downregulated the expression of inflammatory cytokines and the TLR4-IRAK4-NF-κb signaling pathway stimulated by *P. gingivalis* LPS in human periodontal ligament cells (30). Gu et al. documented that berberine had an inhibitory effect on periodontitis in rats, and its putative mechanism of action was attributed to the downregulation of the activity of the P38MAPK/NF-κB pathway mediated by the estrogen receptor (31). Shu et al. found that estrogen may play a significant role in modulating periodontal tissue responses to LPS by reversing the stimulatory effects of LPS on pro-inflammatory cytokine expression (32).

Estrogen and its receptors have exerted significant effects on both immune modulation and inflammatory responses during infection (33). By negatively regulating NF-κB, E2 suppresses the production of pro-inflammatory cytokines and reduces excessive inflammation that can cause tissue damage (33–35). E2 could also modulate type-I interferon responses and adaptive immune responses (33, 36). For instance, E2 shifted macrophages toward M2 repair profile in a variety of disease and injury models (37, 38) and regulated circulating antibody levels to influence the differentiation and function of T cells (39, 40). Our study found that E2 treatment inhibited the expressions of inflammatory cytokines (IL-1, IL-6, and IL-8) induced by *P. gingivalis* LPS. Moreover, RNA sequencing (RNA-seq) data showed that the LPS + E2 group, E2 group, and control group were at similar transcriptional states, including decreased inflammatory response-related pathways. In sum, the activation of estrogen receptors by estrogen or other molecules could downregulate the immune or inflammation response induced by LPS.

On the other hand, the gingival epithelial barrier is the first line of defense against pathogens or metabolites invasion (10). Various transmembrane proteins connect gingival epithelial cells, including tight junctions, adherens junctions, gap junctions, and desmosomes, and they constitute a crucial part of the epithelial barrier (10). The integrity of the barrier plays an important role in maintaining periodontal homeostasis (41). *P. gingivalis* LPS impacted the expression and structural integrity of different cell-cell junctions, resulting in the progression of periodontitis (42, 43). Furthermore, the enhancing effects of estrogen on the epithelial barrier have been preliminarily revealed, such as on the intestinal, dorsal lingual, and esophageal epithelial barrier (20, 21, 44). However, for the gingival epithelial barrier, the effect of E2 on protecting functions from *P. gingivalis* LPS challenge remains unknown. Our results indicated E2 treatment rescued the expression of tight junction-related factors inhibited by *P. gingivalis*-LPS, especially JAM1 and OCLN, both in hGECs and sham/OVX mice to restore the functions of the gingival epithelial barrier, indicating the protective effect of estrogen against *P. gingivalis*-LPS-induced injury to the gingival epithelial barrier.

The causal relationship between the epithelial inflammatory response and epithelial barrier disruption has always been unclear (45). In this study, the RNA-seq data showed that the expression of inflammatory cytokines (IL-1, IL-6, and IL-8) and the activation of inflammatory pathways were decreased in the LPS + E2 group, indicating a decreased inflammatory response. Meanwhile, the gingival epithelial barrier injury was relieved by supplementary E2 treatment. Considering that a lessened invasion of pathogens or metabolites may lead to lower inflammatory responses, we assumed that the relieved epithelial barrier could reduce the inflammatory response by resisting the invasion. The transport and cytokine assays partially supported this hypothesis and showed that the supplementary E2 treatment decreased the transport level and inhibited IL-1β expression in macrophages (lower compartment; Fig. 5B). In addition, lower inflammatory bone resorption was observed in the sham/OVX + LPS + E2 groups compared to the sham/OVX + LPS groups, respectively. These data indicated that the recovered epithelial barrier could reduce the inflammatory response by resisting the invasion of pathogens or metabolites. However, more investigations are still required in the future to reveal their detailed mechanisms and develop more possible therapeutic strategies against periodontitis by targeting this process, such as E2 in this study.

## MATERIALS AND METHODS

### Cell culture and chemicals

Primary hGECs (46) and human monocytic cell line (THP-1) were obtained from the State Key Laboratory of Oral Diseases, National Clinical Research Center for Oral Diseases, West China School of Stomatology, Sichuan University. hGECs were purchased from Otwo Biotech (Shenzhen, China) and grown in Dulbecco’s modified Eagle’s medium (DMEM; Gibco, CA, USA) supplemented with 10% fetal bovine serum (FBS; Gibco, CA, USA). hGECs were all within 10 generations. THP-1 cells were grown in RPMI-1640 medium (Sigma‒Aldrich, MO, USA) with 5% fetal bovine serum. Macrophages were differentiated from THP-1 cells using 4-a-phorbol 12-myristate 13-acetate (30 ng/mL).

*P. gingivalis* LPS was purchased from InvivoGen company (CA, USA) and dissolved in 1 mL of endotoxin-free water to create a 1 mg/mL stock solution for the following use. E2 was purchased from APExBIO company (Houston, USA).

### RNA extraction and quantitative real-time PCR

hGECs were treated with 40 or 80 µg/mL *P*. *gingivalis*-LPS and/or 0.1 or 1 µM E2 and cultured for 18 h (*n* = 3). TRIzol reagent was used to extract the total RNA (Thermo Fisher Scientific, Massachusetts, USA). Purity and concentration were measured by using a spectrophotometer (NanoDrop One; Thermo Fisher Scientific, Massachusetts, USA). PrimeScript TMRT (TAKARA, Shiga, Japan) was used to reverse transcribe RNA to cDNA. In accordance with the manufacturer’s instructions, cDNA was synthesized with PrimeScript RT Master Mix (TaKaRa, Shiga, Japan) and utilized as a template sample for quantitative real-time PCR on a Roche LightCycler 480 Real-Time PCR Detection System (Roche, Basel, Switzerland). The primers used were synthesized by Shenggong Company (Chengdu, China) and were included in Table S1. β-actin was used as the internal reference gene for normalization of gene expression.

### Enzyme-linked immunosorbent assay

hGECs were cultured for 18 h under different treatments (*n* = 3). The culture supernatant was tested using an ELISA kit (IL-1, IL-6, IL-8; MEIMIAN, Jiangsu, China) as directed by the manufacturer. Interpolation from a standard curve was used to calculate the concentrations of IL-1, IL-6, and IL-8.

### Western blotting

hGECs were cultured for 18 h. A total protein extraction kit (SAB, CA, USA) was used to lyse hGECs, and a BCA protein assay kit (Beyotime, Shanghai, China) was used to determine the protein concentration. Protein samples were separated using SDS-PAGE (Beyotime, Shanghai, China) and transferred to a 0.45 m PVDF membrane (Millipore, Shanghai, China) blocked with 5% bovine serum albumin (Biofroxx, German). Anti-OCLN (1:1,000, Affinity, USA), anti-JAM1 (1:1,000, Affinity, USA), anti-β-actin (1:1,000, Affinity, USA), and anti-GADPH (1:1,000, Affinity, USA) antibodies were used to incubate proteins at 4°C overnight. The PVDF membrane was incubated for 1 h with horseradish peroxidase (HRP) -conjugated secondary antibodies (SAB, CA, USA). ECL Western Blotting Substrate (4Abio, Beijing, China) and ChemiDoc MP imaging system (Biorad, CA, USA) were used to detect the immunoreactive bands. ImageJ software (v1.8.0) was used to quantify the data.

### Transwell assay

A 24-well transwell plate (Corning, NK, USA) was used for hGECs culture. The cell density was adjusted to 1 × 10^5^ cells/mL. One milliliter of DMEM with 10% FBS was placed into the bottom compartment. The transwell culture inserts were filled with 100 µL of adjusted hGECs and incubated until they were almost confluent. The medium in the upper chamber was changed to fresh medium with 40 or 80 µg/mL *P*. *gingivalis* LPS (InvivoGen, CA, USA) and/or 0.1 or 1 µM E2 (APExBIO, Houston, USA). After 18 h, the upper compartment was filled with 5 µL of 10 mg/mL FITC-dextran (4.4 kDa, Sigma, USA). The fluorescence signal in the bottom compartment was determined at 485/520 nm after 1 h by the SpectraMax iD5 (Molecular Devices, CA, USA).

### RNA sequencing

The cells were incubated for 18 h after being stimulated with 80 g/mL *P*. *gingivalis* LPS and/or 1 M E2. The cells’ total RNA was then extracted using TRIzol reagent. RNA purification, reverse transcription, library construction, and sequencing were performed by Shanghai Majorbio Biopharm Biotechnology Co., Ltd. (Shanghai, China), according to the manufacturer’s instructions (Illumina, San Diego, CA). A Bioanalyzer (Agilent Technologies) was used to determine RNA quality, and ND-2000 was used to quantify RNA samples (NanoDrop Technologies). The transcriptome library was created using 1 µg of total RNA and the Illumina TruSeqTM RNA sample preparation kit (San Diego, CA). An Illumina NovaSeq 6000 was used for sequencing (2 × 150 bp read length).

### Data processing and bioinformatics analysis

Raw sequencing reads were processed by FastQC and aligned to GRCh38 using STAR. RSEM (v3.1.1) was used to quantify the transcript abundances. PCA was carried out using R software (v3.2.0). DESeq2 (v4.1.2) and ggPlot 2 (v3.4.0) were used to detect DEGs (|log FC| > 1.5 and *P* -adjust < 0.05) and visualization. The KEGG online biological information database (v3.0) was used for the KEGG pathway enrichment analysis of DEGs.

### Animal study

Eight-week-old C57BL/6 female mice (Dashuo Company, Chengdu, China) were housed under specific pathogen-free conditions. The West China School of Sichuan University Ethics Committee approved all animal procedures in this study (WCHSIRB-D-2019–185). The mice (*n* = 48) were sedated with Avertin (0.65 mL/20 g body weight) and underwent bilateral ovariectomy. Six weeks after surgery, the mice were randomly divided into eight groups: (i) OVX; (ii) OVX + E2; (iii) OVX + LPS; (iv) OVX + LPS + E2; (v) Sham; (vi) Sham + E2; (vii) Sham + LPS; and (viii) Sham + LPS + E2. As previously reported, the periodontitis model was created by injecting *P. gingivalis* LPS (20 µg per injection time in 10 µL of PBS total volume, saline as a control) into the gingiva around the palatal first molars (47). Meanwhile, mice in groups (ii), (iv), (vi), and (viii) were intraperitoneally injected with 10 µg/kg/day E2, and mice in the other groups received intraperitoneal injections of saline as a control to explore the function of E2. LPS, E2, and saline injections were administered on the same day and repeated every 2 days for a total of 4 weeks. All mice were sacrificed after 4 weeks of continuous injection.

### Micro-CT analyses

The mandibles were scanned using a CT 50 imaging system (Scanco Medical AG, Bassersdorf, Switzerland) at 10 µm resolution, 70 kV and 114 mA. Three-dimensional image analysis software was used to analyze the bone loss distance. The TV and BV were measured from the reconstructed volume of interest.

### Immunohistochemistry

After fixing in 4% paraformaldehyde for 24 h, samples were decalcified in 10% EDTA (pH = 7.25–7.35) until the probe could extend without resistance. Next, serial 5-μm-thick slices were obtained by cutting the samples embedded in paraffin. Hydrogen peroxide (3%) was used to inhibit any endogenous peroxidase activity. The primary antibodies anti-OCLN (1:1,000, Affinity, USA) and anti-JAM1 (1:1,000, Affinity, USA) were used. To explore the expression of JAM1 and OCLN, three slices from each mouse were analyzed. In each slice, the gingival epithelium areas were first identified by histologically distinguishing the interface between the epithelial basal layer and the connective tissue; second, immune-positive areas of JAM1 or OCLN in five randomly selected areas within the gingival epithelium region were further calculated under 400 × magnification.

### Transport and cytokine assays

The model imitated Abe’s transport assay model (37). Briefly, the hGECs were adjusted to 1 × 10^5^ cells/mL. A 24-well transwell plate was used. One milliliter of DMEM with 10% FBS was placed into the bottom compartment. The culture inserts were filled with 100 µL of adjusted cells. The cells were incubated until they were almost confluent. The medium in the upper chamber was changed to fresh medium with 40/80 µg/mL *P*. *gingivalis* LPS and/or 0.1/1 µM E2. The cells were incubated for 18 h, and the medium was removed from both compartments. The upper compartment was placed in a newly prepared culture plate containing macrophages. *P. gingivalis* LPS (80 µg/mL) was added to the upper compartment, and medium without LPS was added to the lower compartment (Fig. 4A). The medium supernatants were assayed for IL-1β using ELISA, as described above, after incubating for 2 h.

### Statistical analysis

Analysis of variance (ANOVA) with Tukey’s multiple comparison test was conducted using GraphPad Prism (v8.2.1, GraphPad Software Inc, San Diego, CA, USA), and results with *P* < 0.05 were considered statistically significant. All data are shown as the mean ± SD.

## Data Availability

The data presented in this study are available on request from the corresponding author. The RNA-seq data reported in this paper have been deposited in the Genome Sequence Archive in the National Genomics Data Center, China National Center for Bioinformation/Beijing Institute of Genomics, Chinese Academy of Sciences (no. HRA004287).

## SUPPLEMENTAL MATERIAL

Supplemental material iai.00410-24-s0001.docx Fig. S1 and S2; Table S1.

ASM does not own the copyrights to Supplemental Material that may be linked to, or accessed through, an article. The authors have granted ASM a non-exclusive, world-wide license to publish the Supplemental Material files. Please contact the corresponding author directly for reuse.
