# Supplementary material for: Estrogen prevented gingival barrier injury from Porphyromonas gingivalis lipopolysaccharide
Source: Infect Immun. 2025 Feb 20;93(3):e00410-24. doi: 10.1128/iai.00410-24 (PMC11918251; doi:10.1128/iai.00410-24)
Supplement: Supplemental material — Fig. S1 and S2; Table S1. [file iai.00410-24-s0001.docx]

**Supplementary materials**

**
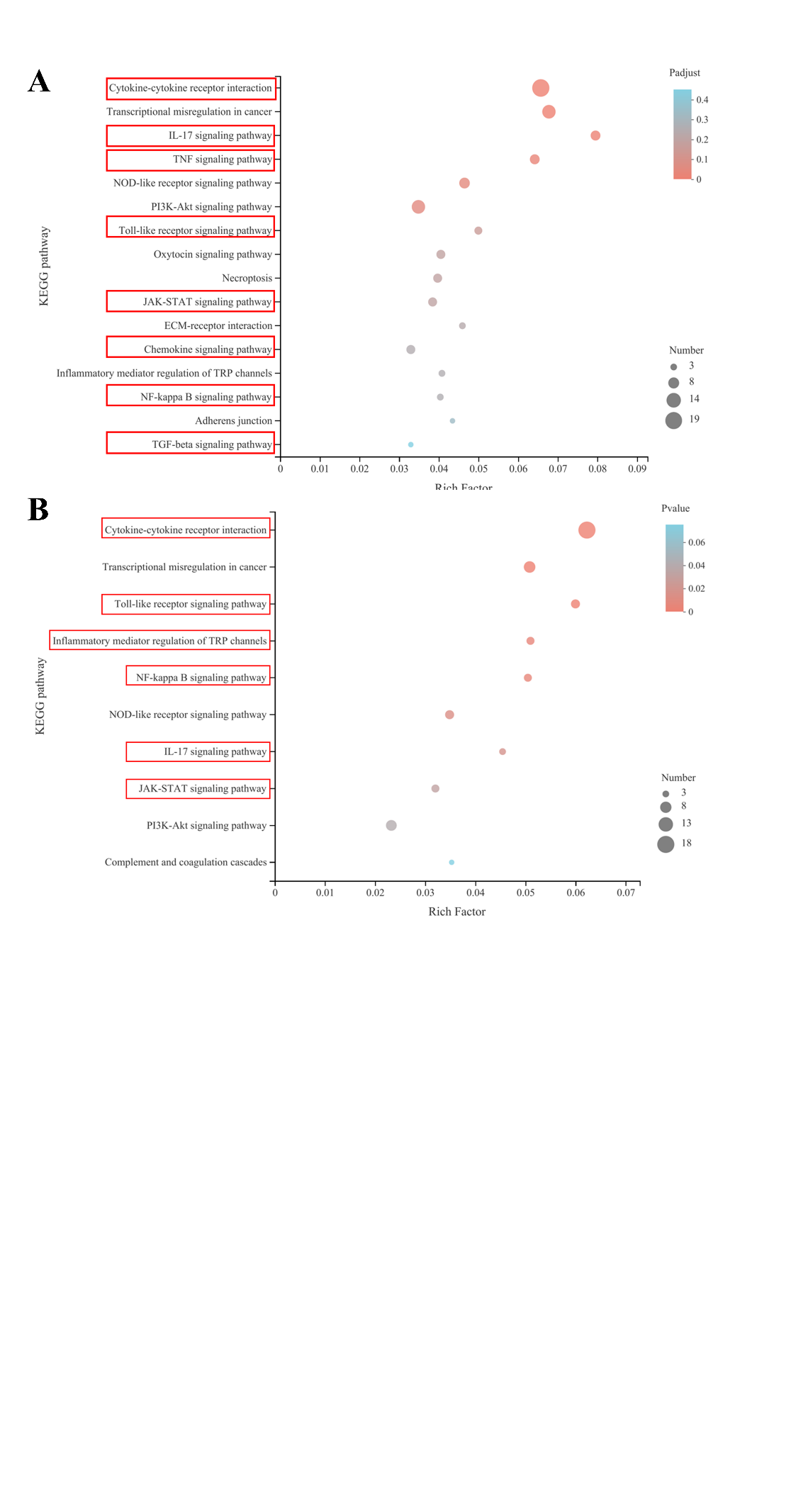
**

**
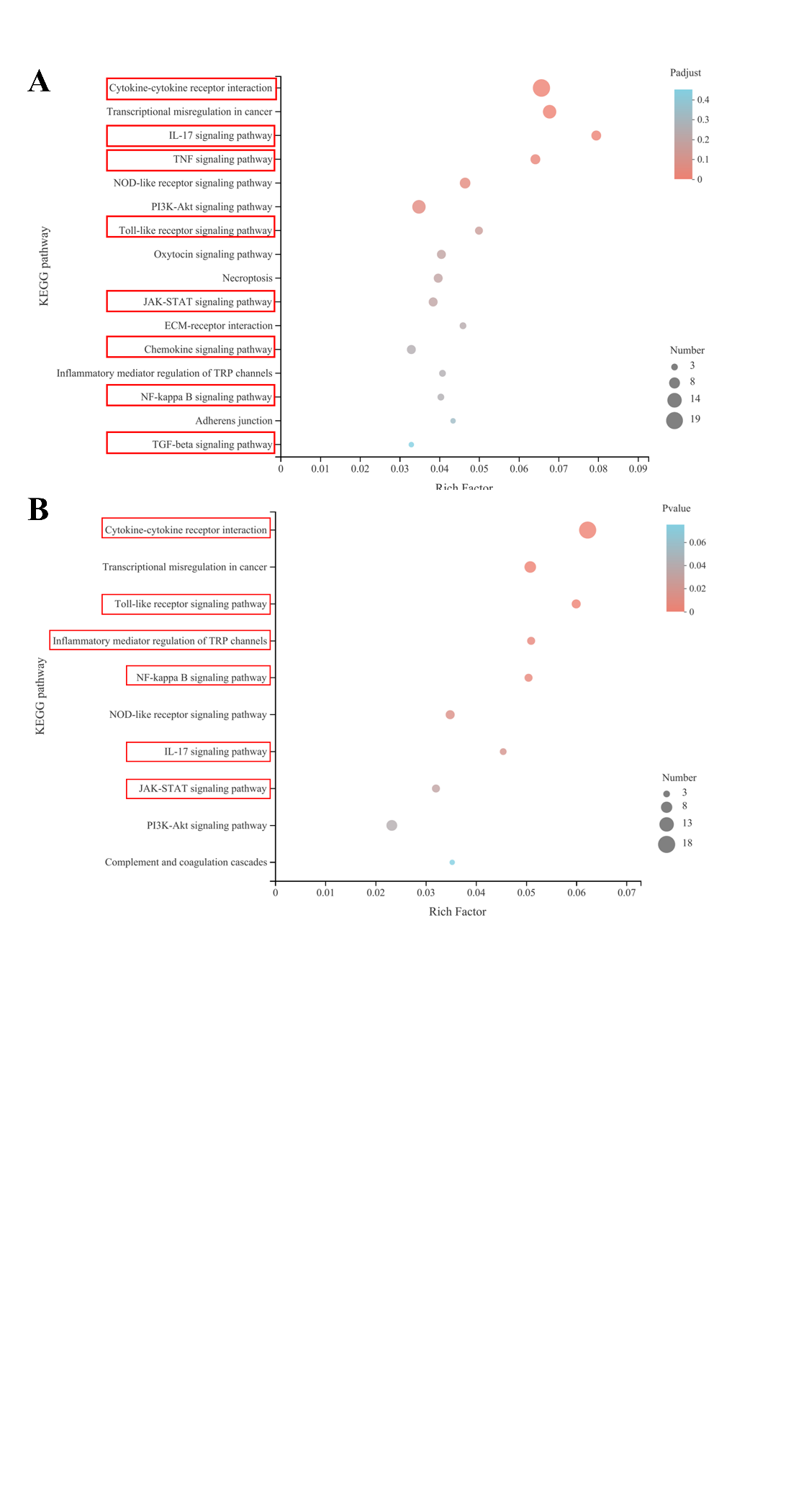
**

**Figure S1.** KEGG pathway analysis of differentially expressed genes. (A) Upregulated genes in the LPS group compared with the control group. (B) Downregulated genes in the LPS+E2 group compared with the LPS group. Red box: inflammation-related pathways


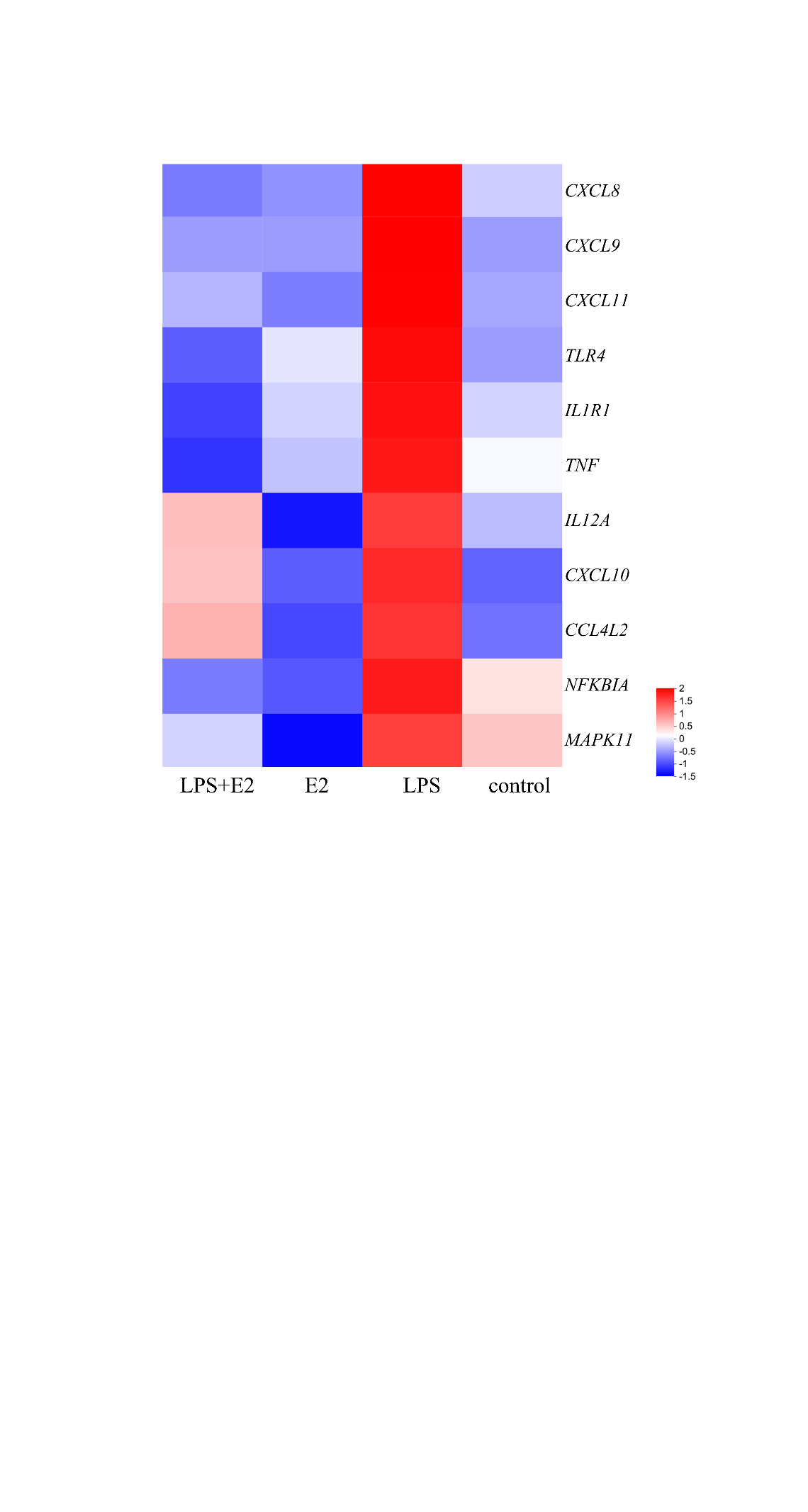


**Figure S2.** Heatmap of the expression levels of inflammation-related genes in each group.

**Table S1** **Primer pairs used in this study**

| Target genes | Primers |
| --- | --- |
| *IL-1b*-F | 5’-CACTATCGGCGGTTACGAAT-3’ |
| *IL-1b*-R | 5’-CAATTTGGAGCAAGTCAGCA-3’ |
| *IL-6*-F | 5’-TCTCCACAAGCGCCTTCG-3’ |
| *IL-6*-R | 5’-CTCAGGGCTGAGATGCCG-3 |
| *IL-8*-F | 5’-ATGACTTCCAAGCTGGCC-3’ |
| *IL-8*-R | 5’-CAAGAGAGCCACGGCCAG-3 |
| *JAM*-F | 5’-AAGGCAAGGGTTCGGTGTA -3 |
| *JAM*-R | 5’-CACTGGATGAGAAGGTGACG -3 |
| *OCLN*-F | 5’-GCACGTTCGACCAATGCTCT -3’ |
| *OCLN*-R | 5’-AGATGCCGTTCCATAGGGT-3’ |
| *β-actin*-F | 5’-CCTGGCACCCAGCACAAT-3 |
| *β-actin*-R | 5’-GGGCCGGACTCGTCATAC-3 |
